# Skin biology and ageing

**DOI:** 10.1002/ski2.258

**Published:** 2023-06-11

**Authors:** Ewan A. Langan, George W. M. Millington

**Affiliations:** ^1^ Department of Dermatology University of Lübeck Lübeck Germany; ^2^ Dermatological Sciences University of Manchester Manchester UK; ^3^ Dermatology Department Norfolk and Norwich University Hospital Norwich UK; ^4^ Norwich Medical School Norwich UK

Over the last few decades there has been a dramatic increase in our understanding of the pathophysiology of a range of common, chronic inflammatory skin diseases, such as psoriasis and atopic eczema (dermatitis). Indeed, this knowledge has already enabled the rapid expansion of our range of dermatological treatments, which now includes a first‐in‐class allosteric tyrosine kinase 2 inhibitor for psoriasis,^1^ a range of oral JAK inhibitors for atopic dermatitis and alopecia areata^2^, ^3^ and a topical JAK inhibitor for vitiligo.^4^ There have also been major advances in the treatment of skin cancers, with the dawn of immune checkpoint inhibitors and the development of novel topical treatments for actinic keratoses.^5^

However, the maintenance of skin health and the prevention of skin disease should not be neglected. Indeed, when *Skin Health and Disease* was launched, a key aim of the journal was to deepen our understanding of skin biology in order to prevent skin disease and hair disorders. We are particularly interested in basic and clinical research examining skin health across the life cycle and the effects of both intrinsic and extrinsic ageing on skin biology and function.

To this end, we are pleased to announce the launch of our third special edition which will centre on Skin Biology and Ageing. We are delighted to welcome Professor Rachel Watson (Head of the Skin Research Institute of Singapore) and Abigail Langton (Lecturer in Ethnic Skin at the University of Manchester) as guest editors. We are keen to receive submissions which focus on Skin Biology and Ageing in the broadest sense and focussing on all skin types, especially interdisciplinary research. We also have a range of article categories to ensure that authors can select the most appropriate way to publish their work. If you wish to discuss a submission with us, or with any of the guest editors, please email shd@bad.org.uk.

## CONFLICT OF INTEREST STATEMENT

Ewan A. Langan is the Editor in Chief of *Skin Health and Disease*. George W. M. Millington is the Deputy Editor of *Skin Health and Disease.*

## AUTHOR CONTRIBUTIONS

**Ewan A. Langan**: Writing – original draft (Lead); Writing – review & editing (Equal). **George W. M. Millington**: Writing – original draft (Supporting); Writing – review & editing (Equal).

## FUNDING INFORMATION

This editorial received no specific grant from any funding agency in the public, commercial, or not‐for‐profit sectors.

## ETHICS STATEMENT

Not applicable.

## Data Availability

Data sharing not applicable to this article as no datasets were generated or analysed during the current study.
